# Whole-genome sequence of non-rhizobial strain *Tardiphaga* sp. 709 isolated from the root nodule of *Astragalus inopinatus* Borris., growing on the Kamchatka Peninsula

**DOI:** 10.1128/mra.00923-24

**Published:** 2024-12-17

**Authors:** Polina Guro, Irina Kuznetsova, Anna Sazanova, Andrey Belimov, Vera Safronova

**Affiliations:** 1 All-Russia Research Institute for Agricultural Microbiology (ARRIAM), St.-Petersburg, Russia; The University of Arizona, Tucson, Arizona, USA

**Keywords:** Kamchatka Peninsula, whole-genome sequencing, non-rhizobial endophyte

## Abstract

We report the whole-genome sequence of the non-rhizobial endosymbiotic bacteria *Tardiphaga* sp. strain 709, which was isolated from the root nodule of *Astragalus inopinatus* Borris. on the Kamchatka Peninsula, Russia. The genome consists of one chromosome and one plasmid with a total length of 6,359,564 bp and 61.5% of GC content.

## ANNOUNCEMENT

*Tardiphaga* is a genus belonging to the *Nitrobacteraceae* family within the class *Alphaproteobacteria*. Members of this genus are widely distributed and can be found in diverse environments, including root nodules of leguminous plants, arctic glacier water, and soil samples (1–3). The Tardiphaga genus bacteria are characterized as Gram-negative, slow-growing, rod-shaped, and aerobic. Some strains exhibit phototrophic capabilities and tolerance to heavy metals (4–6). Sequencing the genome of *Tardiphaga* sp. strain 709 may identify genetic adaptations for symbiosis with leguminous plants, enhancing our understanding of endosymbiosis and benefiting agricultural and ecological research. Strain 709 was isolated from the root nodule of the leguminous plant *A. inopinatus*. The plant sample was collected on the Kamchatka Peninsula in the Russian Federation (55.925500°N, 159.716370°E, h = 40). A single nodule was sterilized for 1 minute in 70% ethanol, rinsed, homogenized in sterile tap water, and plated on a YMSA agar plate (7). Colonies appeared after 6 days of incubation at 28°C. A single colony was re-cloned twice on the medium to obtain a pure culture. Genomic DNA without size selection was isolated using the DNeasy Blood & Tissue kit (Qiagen, Germany) according to the manufacturer’s recommendations from a pure culture grown in liquid R2A broth at 28°C for 48 hours. The library was prepared using the SQK-LSK109 ligation sequencing kit and the EXP-NBD104 native barcoding expansion 1–12 kit (Oxford Nanopore Technologies [ONT], UK), skipping the DNA shearing step and sequenced using a MinION sequencer with Flow Cell R.9.4.1 (ONT, UK). Basecalling was performed using Guppy (v5.0.1, ONT, UK) with the high-accuracy model. Sequencing yielded a total of 1,015,946,059 bp across 103,589 reads, with an average read length of 9,807 bp and approximately 159-fold coverage. Quality control (QC) of the raw reads was performed using NanoStat (v1.6.0) (8). The ONT reads were then filtered using the Filtlong tool (v0.2.1, https://github.com/rrwick/Filtlong), with options: --min_length 1000; --keep_percent 95. QC after filtering results in 96,457 reads and an average read length of 15,991 bp. *De novo* assembly was performed using Flye (v2.9), resulting in two circular contigs: a chromosome of 6,259,763 bp and a plasmid of 99,801 bp (9). The total genome size was 6.4 Mbp with 153× coverage, and no specific start point was designated. To generate consensus sequences, the resulting assembly was polished using a Medaka (v1.8.0, https://github.com/nanoporetech/medaka) using raw reads. Default parameters were used for all software unless otherwise noted. Assembly statistics were provided by QUAST (v5.0.2) (10). The GC content was 61.49% and the N_50_ value of the assembled genome was 6,259,763 bp. The taxonomic rank was determined by 16S rRNA gene (*rrs*) BLASTn comparison and average nucleotide identity (ANI) analysis calculated using JspeciesWS (v 0.93.1) (11, 12). Strain 709 was most closely related to the type strain *T. robiniae* R-45977T (GCF_013359755.1; NR_117178.1) with 93.62% average nucleotide identity and 99.57% *rrs* identity. On this basis, strain 709 was assigned to *Tardiphaga* sp. and may represent a new species. Annotation of the assembly using PGAP (v 6.6) resulted in 5,891 protein-coding genes and 58 RNA genes (6 rRNAs, 48 tRNAs, and 4 ncRNAs) (13).

## Data Availability

All data are available at GenBank under BioProject accession number PRJNA1018331. The BioSample accession number is SAMN37433447. The raw MinION data can be found under the number SRR26465730 and the assembly accession number NZ_CP135529.1-NZ_CP135530.1.
